# Health and fitness trends in Iran for 2024: A cross-sectional study

**DOI:** 10.3934/publichealth.2023053

**Published:** 2023-10-07

**Authors:** Alexios Batrakoulis, Saeid Fatolahi, Farnaz Dinizadeh

**Affiliations:** 1 Department of Physical Education and Sport Science, University of Thessaly, Trikala, Greece; 2 Department of Exercise Sciences, Tarbiat Modares University, Tehran, Iran.; 3 Department of Physical Education, Tabriz Branch of Islamic Azad University, Tabriz, Iran.

**Keywords:** Iran, fitness survey, trends, top programs, top services, ACSM survey

## Abstract

The health and fitness sector is evolving and appears to be an important field not only for consumers but also for gym operators/managers, exercise professionals, training providers and educators with great potential worldwide. Our aim of this cross-sectional survey was to investigate the most attractive health and fitness trends in Iran for the first time and to observe any potential differences with the recent results reported in other regions. A national online survey was conducted, using the methodology of similar international surveys conducted by the American College of Sports Medicine since 2007. In total, a web-based questionnaire was sent to 7158 professionals who worked in the Iranian health and fitness industry. A total of 408 responses were collected with a response rate of 5.7%. The 10 most important health and fitness trends in Iran for 2024 were strength training with free weights, exercise for weight loss, group training, low-cost and budget gyms, dance-based workouts, outdoor activities, Pilates, bodyweight training, core training and aquatic exercise. The present findings are not fully aligned with those reported for the top health and fitness trends internationally, showing that trends related to technology and health are not yet popular nationwide. Such outcomes may support all industry stakeholders with making important business decisions, professional development opportunities and innovative concepts to enhance customer engagement through positive exercise experiences.

## 1. Introduction

The global health and fitness industry is currently experiencing enormous evolution, demonstrating a substantially growth in terms of all industry stakeholders, such as operators, personnel, consumers and enterprises [1]. This particular industry offers various fitness services and products and has recently passed through a very challenging 3-year period not only due to the coronavirus pandemic (COVID-19) but also an almost 10-year economic crisis seriously impacting emerging and developing markets as those mainly observed in the Asian region [2]. However, recent data indicate the powerful role of the health and fitness industry in public health and economy globally, through popular trends among entrepreneurs, employees and exercisers [3]. In Iran, a medium to small gym size suits the cost saving strategy for operators and also better fits into customers' needs, representing a large majority of the market nationwide. Nevertheless, sports complexes are currently on the rise, creating an important business field for the local economy by promoting innovative fitness services with great potential for growth in the future [4]. Such large, multi-purpose facilities offer a luxurious and relaxing environment, combining outdoor sports fields and indoor areas for fitness. However, the increasing prevalence of sedentarism and excess weight appear to be some major public health concerns not only in Iran but also worldwide. More specifically, 32% of the adult population are physically inactive, 65% are overweight and 26% have obesity in Iran. Such national epidemiological data are similar to those reported in the Western world [5],[6]. On the other side, regular physical activity and exercise seem to be a dynamic tool for tackling the inactivity and obesity epidemics that promote numerous chronic diseases among all ages, races/ethnic groups and sexes [7],[8].

Iran is a developing country with a lower-middle income economy and is the ninth most populated country in Asia [9]. The prevalence of the most prevalent lifestyle-related chronic diseases is systematically increasing nationwide, harmfully affecting public health status among adults [10]. In particular, various cardiometabolic health-related issues such as inactivity, overweight/obesity, insulin resistance, prehypertension/hypertension and dyslipidemia remarkably influence the vast majority of citizens in Iran [10]. Importantly, the Iranian health and fitness industry is currently increasing its size, developing more facilities, recruiting more employees and attracting more customers. However, there are no fitness chains and private business models in fitness centers do not exist in a structured and well-defined way [4]. Importantly, the most impactful factors in decision-making and customer attraction in Iranian fitness centers are social features, access options, health-related outcomes and demographic characteristics [4]. It is noteworthy that according to a population based cross-sectional study, various environmental, social and service factors may play a key role in customer attraction targeting to health promotion behaviors among females in Iran [11] In general, there are no available data for the current status of the Iranian health and fitness industry. Therefore, important information regarding the market size as well as growth and penetration rates at the national level are unknown. Such an observation may underline the rationale of further investigation in this particular research area, focusing on emerging fitness markets.

A worldwide survey on health and fitness trends has been conducted on a yearly basis by the American College of Sports Medicine (ACSM) since 2006. Such a research approach aims to determine the most attractive and respectable training concepts, including modalities, products and services among all industry stakeholders [12]–[28]. In particular, surveys of this kind may help consumers consider engaging in evidence-based practices, aiming to experience safe, enjoyable and effective fitness services. In addition, such research projects may support gym operators, practitioners and educators to focus on new opportunities, targeting a new landscape linked to research-based outcomes and implications [28]. The current status of trends in the Iranian health and fitness industry has not been yet studied. Considering this, it is obvious that the lack of in-depth investigation of the most popular health and fitness trends creates a gap between science and practice nationwide. On the contrary, extensive research has been previously conducted by the ACSM with a focus on various regions, collecting data and providing comparative analyses at the international level [29]–[33]. Importantly, numerous regional and national cross-sectional studies have replicated the ACSM's methodology, disseminating relevant outcomes in order to create awareness of the top trends in the health and fitness industry internationally [34]–[42].

The present cross-sectional survey is the first study aiming to collect data from Iran and intending to compare the findings with those reported for other countries and regions [28],[33]. Only data from China have already published from the Asian region in the past [30]–[32],[40]. Taking this into account, such a study will provide novel evidence on health and fitness trends from another Asian country and this is important given that this particular region is characterized by various emerging national markets. Hence, our objectives of this observational study were a) to detect the most popular health and fitness trends in Iran for 2024 and b) to compare these trends between regions around the globe. In summary, the present study may support all stakeholders to help decision makers on how to tackle the obesity and inactivity epidemics through engaging exercise experiences and policies targeting the health and fitness needs of the masses nationwide.

## 2. Materials and Methods

### 2.1. Study design

A cross-sectional study of health and fitness trends was conducted, applying a web-based survey and a descriptive approach. The present observational study used the same methodology with relevant surveys conducted by the ACSM. More specifically, the present survey used similar criteria to those that have been widely included in relevant national [34]–[42], regional [29]–[33] and worldwide [12]–[28] surveys of health and fitness trends since 2006. In this study, data collection was made from a local survey aiming to present the main findings not only at the national but also the international level through a comparison with those recently reported for other countries and regions [28],[33]. Briefly, the survey was created to examine the health and fitness trends (not fads) that are considered attractive because of their impactful role in the local industry while showing raised popularity among practitioners based in Iran, involving various fitness services with a broad spectrum of consumers in several exercise settings. Hence, the introduction of the survey included a distinction between “fad” and “trend” in accordance with the dictionary, aiming to support participants to identify the difference between these two key terms as previously articulated [12]–[28].

### 2.2. Sample recruitment and inclusion criteria

Adults aged 18 and older with any occupational role, professional experience and education level, work status and annual salary in the Iranian health and fitness industry were considered eligible participants and included in the study. Databases of contacts of local faculties of physical education and sports sciences across the country were mainly used to recruit participants for this survey. Moreover, the national registry of exercise professionals was also used to collect data. The online survey was sent electronically to 7,158 individual contacts in total. All contacts were people highly involved in the Iranian health and fitness industry under various occupational roles. Likewise, several posts on social media and relevant websites were made in collaboration with all involved parties to increase the awareness of the survey nationwide.

### 2.3. Data collection tool

The present study used a widely established methodology developed by the ACSM. In particular, a group of technical experts with extensive experience in health and fitness as practitioners and/or educators was recruited to identify a list of fitness trends [28]. This group (N =30) of industry experts and academics from all over the world was involved in a pilot study to finalize the list of prospective health and fitness trends from which the web-based survey was developed after verifying the consistency of the questionnaire and modifications were applied accordingly [39]. The reliability of the questionnaire was assessed by calculating the alpha–Cronbach's coefficient, showing an acceptable value (0.76). Hence, a web-based questionnaire using an online survey platform (Google Forms) was developed, including 47 related trends that were retrieved from both several sources and experts' personal experiences. Each trend included in the questionnaire was accompanied by a brief description, helping respondents understand better each option as previously reported [28]. A 10-point Likert scale ranging from 1 (least likely to be a trend) to 10 (most likely to be a trend) was used to evaluate the potential trends as previously described [28]. The questionnaire required less than 15 minutes to be completed and included several demographic questions regarding various characteristics, such as gender, age, region, education, certification(s), occupation, experience, work status, work setting and annual salary. The questionnaire was provided in Persian with no changes from the original English version designed by the ACSM. The Iranian authors (S.F. and F.S.) of the present study was in charge of translation into local language. Afterwards, native/bilingual English speakers, with an academic background in exercise science reviewed the draft and revised it where needed, aiming to be 100% correct compared to the original English version.

### 2.4. Recruitment and study period

The survey was conducted electronically from January 2023 to March 2023 (12 weeks). Financial or material incentives were not offered to attract respondents to the study. All database contacts were received numerous email reminders during the 3-month study period. Participants were asked to fill out the questionnaire anonymously and to sign an informed consent letter at the beginning of the process. All required details concerning the research objectives, confidentiality of information and the right to withdraw the participation in the survey were included on the first page of the web-based questionnaire.

### 2.5. Data analysis

The present explanatory study was methodologically based on numerous similar surveys extensively conducted by the ACSM and international partners [12]–[42], and thus the data collected were analyzed using quantitative methods. Each item of data was scrutinized for accuracy. Descriptive and inferential statistics, such as percentage frequency distributions, means and standard deviations were used to report the outcomes. Outliers and the normality of distributions were checked using frequency distributions. All descriptive analyses were performed using the IBM SPSS Statistics 26.0 software (IBM Corp., Armonk, NY, USA).

## 3. Results

In total, the national online survey collected 408 responses, which represents a return rate of 5.7%. According to the demographic characteristics (Table 1), respondents from all provinces of Iran (55.6% females and 44.4% males) with diverse backgrounds, occupations and experience levels participated in the study. More specifically, 53.2% of respondents had over 10 years of professional experience in the industry and 18.6% had over 20 years of experience. Furthermore, 62% of respondents currently work as practitioners under various occupational roles, mostly as part-time personal trainers (17.5%), while 87.7% of participants held at least a bachelor's degree in exercise science or a related field. Part-time work and number one career choice were stated by 58.3% and 80.9%, respectively. Lastly, 32.4% of participants reported an annual salary between $900 and $1500 USD. All health and fitness trends included in the survey were ranked from highest (most popular trend) to lowest (least popular trend) mean score and are illustrated in Table 2. A comparison of the top 20 health and fitness trends among Iran and other countries/regions is shown in Table 3. Trends were also categorized in the following six groups, as reported elsewhere [42]: trends related to i) fitness professionals, ii) fitness activities, iii) training modalities, iv) programs oriented to specific populations, v) technology and iv) health. Table 4 presents a grouped approach of the comparative analysis of the top 20 health and fitness trends in Iran and the world.

**Table 1.** Demographics of the survey respondents in Iran.

**Project**		**N**	**%**
*Gender*			
	Female	227	55.6
	Male	181	44.4
*Age (years)*			
	18–21	15	3.7
	22–34	211	51.7
	35–44	143	35.0
	45–54	32	7.8
	55–64	7	1.7
	>65	0	0
*Region (Provinces)*			
	Northern	52	12.7
	Southern	28	6.8
	Western	135	33.0
	Eastern	29	7.1
	Central	164	40.1
*Education*			
	Bachelor's degree	88	21.6
	Master's degree	176	43.1
	Doctoral degree	94	23.0
	Vocational	50	12.3
*Certification(s)*			
	Fitness instructor	159	39.0
	Group exercise instructor	28	6.9
	Personal trainer	39	9.5
	Pilates teacher	32	7.8
	Yoga teacher	19	4.7
	Aqua fitness instructor	22	5.4
	Health coach	17	4.2
	Exercise physiologist	64	15.6
	Clinical exercise physiologist	8	2.0
	Not currently certified	13	3.1
	Other	7	1.8
*Primary profession*			
	Personal trainer (part-time)	71	17.5
	Group exercise instructor	47	11.5
	Owner/operator	45	11.0
	Exercise physiologist	43	10.5
	Vocational educator/tutor	37	9.0
	Personal trainer (full-time)	32	8.0
	Undergraduate student	25	6.1
	University/college professor	20	5.0
	Physical education teacher	17	4.1
	Pilates teacher	16	4.0
	Graduate student	15	3.6
	Yoga teacher	13	3.1
	Health/wellness coach	8	2.0
	Allied healthcare professional	7	1.7
	Gym manager	6	1.5
	Other	3	0.7
	Clinical exercise physiologist	3	0.7
*Experience (years)*			
	0–1	17	4.2
	1–3	16	3.9
	3–5	43	10.5
	5–7	49	12.0
	7–9	66	16.2
	10–20	141	34.6
	>20	76	18.6
*Work status*			
	Full-time	170	41.7
	Part-time	238	58.3
*Career choice*			
	First job	330	80.9
	Second job	60	14.7
	Third job	18	4.4
*Work setting*			
	Private practice/own business	127	31.1
	University facility	62	15.1
	Vocational training provider	61	15.0
	Boutique fitness studio	61	15.0
	Commercial fitness center	28	6.8
	National association	26	6.4
	Corporate fitness facility	16	4.0
	Community-based facility	8	2.0
	Medical fitness center	7	1.7
	Other	7	1.7
	Hospital/medical center	2	0.5
	Supplier	2	0.5
	Sport tourism facility	1	0.3
*Annual salary (USD)*			
	≤900	46	11.1
	901–1500	132	32.4
	1501–2100	75	18.4
	2101–3000	80	19.7
	≥3001	75	18.4

**Table 2.** Comprehensive ranking of future health and fitness trends in Iran.

**#**	**Trend**	**Score**
1	Strength training (free weights)	7.19 ± 2.06
2	Exercise for weight loss	6.90 ± 2.20
3	Group training	6.78 ± 2.09
4	Low-cost and budget gyms	6.64 ± 2.50
5	Dance-based workouts	6.58 ± 2.42
6	Outdoor activities	6.17 ± 2.23
7	Pilates	6.12 ± 2.00
8	Body weight training	6.10 ± 2.17
9	Core training	5.91 ± 2.16
10	Aquatic exercise	5.84 ± 2.33
11	Boutique fitness studios	5.79 ± 2.49
12	Licensure for fitness professionals	5.68 ± 2.54
13	Yoga	5.60 ± 2.22
14	Children and exercise	5.64 ± 2.35
15	Resistance band training	5.53 ± 2.03
16	Walking/running/cycling clubs	5.52 ± 2.36
17	Functional fitness training	5.50 ± 2.00
18	High intensity interval training	5.47 ± 1.99
19	Personal training	5.39 ± 2.29
20	Stretch-based training	5.33 ± 1.98
21	Employing certified fitness professionals	5.33 ± 2.88
22	Small group training	5.31 ± 1.98
23	Plyometric training	5.13 ± 1.94
24	High-intensity functional training	5.10 ± 2.02
25	Outcome measurements	5.08 ± 2.32
26	Home exercise gyms	5.06 ± 2.31
27	Wearable technology	5.01 ± 2.48
28	Post rehabilitation classes	4.00 ± 2.24
29	Fitness programs for older adults	4.97 ± 2.10
30	Online personal training	4.94 ± 2.31
31	Online live and on-demand exercise classes	4.90 ± 2.18
32	Exercise is medicine	4.88 ± 2.15
33	Circuit training	4.85 ± 1.99
34	Health/wellness coaching	4.82 ± 2.18
35	Mobile exercise apps	4.78 ± 2.22
36	Lifestyle medicine	4.78 ± 2.14
37	Long-term youth development	4.60 ± 2.22
38	Medicine ball training	4.59 ± 2.00
39	Mind-body movement	4.58 ± 2.10
40	Electrical muscle stimulation training	4.42 ± 2.28
41	Mobility/myofascial devices/rollers and recovery	4.31 ± 2.09
42	Clinical integration/medical fitness	4.30 ± 2.26
43	Virtual reality exercise training	4.22 ± 2.14
44	Pre-and post-natal fitness	3.96 ± 2.18
45	Balance and stabilization training	3.94 ± 1.82
46	Worksite health promotion programs	3.75 ± 2.31
47	Worker incentive programs	3.64 ± 2.31

Note: Scores are expressed as mean values ± standard deviation.

Briefly, strength training with free weights was selected as the most popular trend in the Iranian health and fitness industry for 2024. Specifically, eight trends related to fitness activities (#1 strength training with free weights, #4 low-cost and budget gyms, #5 dance-based workouts, #6 outdoor activities, #7 Pilates, #8 bodyweight training, #9 core training and #10 aquatic exercise), one related to programs oriented to specific populations (#2 exercise for weight loss), and one related to training modalities (#3 group exercise) are included in the top 10 most attractive health and fitness trends nationwide. No trends related to technology and health were ranked among the top 20 most attractive options nationwide. A grouped comparative analysis of the top 10 health and fitness trends based on age, experience, career choice and annual salary did not show important differences. However, female and male respondents selected group training and strength training with free weights as the number one trend, respectively. Comparing the top 20 Iranian health and fitness for 2024 with the worldwide ones published by the ACSM for 2023, there are 10 trends that are included only in the global list (wearable technology, fitness programs for older adults, functional fitness training, employing certified fitness professionals, circuit training, home exercise gyms, exercise is medicine, lifestyle medicine, health/wellness coaching and mobile exercise apps). Similarly, comparing the top 10 Iranian options with those observed in other regions, such as Australia, Brazil, Europe, Mexico and the United States, several aforementioned health and fitness trends present at the international level were not included in the Iranian top selections.

**Table 3.** Comparative analysis of the top 20 health and fitness trends among Iran and other countries/regions.

**#**	**Iran**	**Australia** [33]	**Brazil** [33]	**Europe** [33]	**Mexico** [33]	**United States** [33]	**World** [28]
1	Strength training (free weights)	Fitness programs for older adults^2^	Personal training	Body weight training	Exercise for weight loss	Wearable technology	Wearable technology
2	Exercise for weight loss	Functional fitness Training	Exercise for weight loss	Exercise for weight loss	Personal training	Strength training (free weights)	Strength training (free weights)
3	Group training	Strength training (free weights)	Fitness programs for older adults^2^	Personal training	Functional fitness training	Body weight training	Body weight training
4	Low-cost and budget gyms^1^	Group training	Functional fitness training	Fitness programs for older adults^2^	Aerobic training	Fitness programs for older adults^2^	Fitness programs for older adults^2^
5	Dance-based workouts^1^	Employing registered Exercise professionals	Body weight training	Functional fitness training	Sport-specific Training	Outdoor activities	Functional fitness training
6	Outdoor activities	Wearable technology	Strength training (free weights)	High-intensity Interval training	Outdoor fitness Activities	Functional fitness Training	Outdoor activities
7	Pilates	Pilates	Employing certified Fitness professionals	Boutique fitness studios	Children and exercise	High-intensity interval training	High-intensity interval training
8	Body weight training	Outdoor activities	Outdoor activities	Circuit training^2^	Body weight training	Exercise for weight loss	Exercise for weight loss
9	Core training	Personal training	Lifestyle medicine	Exercise is medicine	Healthy diet	Employing certified fitness professionals	Employing certified fitness Professionals
10	Aquatic exercise^1^	Body weight training	High-intensity interval training	Employing certified fitness Professionals	Multidisciplinary work teams	Personal training	Personal training
11	Boutique fitness studios	Exercise is medicine	Online personal Training	Strength training (free weights)	Strength training (free weights)	Core training	Core training
12	Licensure for fitness Professionals	Inclusive exercise Services	Small group training^2^	Wearable Technology	Training and feeding programs	Circuit training^2^	Circuit training^2^
13	Yoga	High intensity interval training	Health/wellness Coaching	High-intensity Functional training	Worksite health promotion programs	Home exercise gyms	Home exercise gyms
14	Children and exercise	Small group training^2^	Group training	Outdoor activities	outcome Measurements	Group training	Group training
15	Resistance band training^1^	Yoga	Outcome measurements	Clinical integration / medical fitness	Circuit training^2^	Exercise is medicine	Exercise is medicine
16	Walking/running/cycling clubs^1^	Core training	Post rehabilitation classes	Small group Training^2^	Group training	Lifestyle medicine	Lifestyle medicine
17	Functional fitness training	Lifestyle medicine	Home exercise gyms	Children and exercise	Small group training^2^	Yoga	Yoga
18	High intensity interval training	Exercise for weight loss	Circuit training^2^	Licensure for fitness Professionals	Employing graduates	Licensure for fitness professionals	Licensure for fitness professionals
19	Personal training	Post rehabilitation classes	Wearable technology	Pilates	High intensity interval training	Mobile exercise apps	Health/wellness coaching
20	Stretch-based training^1^	Health/wellness coaching	Core training	Group training	Combat sports training	Health/wellness coaching	Mobile exercise apps

Note: ^1^appearance only in Iran, ^2^non-appearance in Iran.

**Table 4.** A grouped comparative analysis of the top 20 fitness trends in Iran and the world.

**#**	**Iran**	**#**	**World** [28]
*Trends related to fitness professionals:*	
12	Licensure for fitness professionals	9	Employing certified fitness professionals^2^
		18	Licensure for fitness professionals
*Trends related to fitness activities*	
1	Strength training (free weights)	2	Strength training (free weights)
4	Low-cost and budget gyms^1^	3	Body weight training
5	Dance-based workouts^1^	5	Functional fitness training^2^
6	Outdoor activities	6	Outdoor activities
7	Pilates	7	High-intensity interval training
8	Body weight training	11	Core training
9	Core training	12	Circuit training^2^
10	Aquatic exercise^1^	13	Home exercise gyms^2^
11	Boutique fitness studios	17	Yoga
13	Yoga		
15	Resistance band training^1^		
16	Walking/running/cycling clubs^1^		
17	Functional fitness training		
18	High-intensity interval training		
20	Stretch-based training^1^		
*Trends related to training modalities:*	
3	Group training	10	Personal training
19	Personal training	14	Group training
		19	Health/wellness coaching^2^
*Trends related to programs oriented to specific populations:*	
2	Exercise for weight loss	4	Fitness programs for older adults^2^
14	Children and exercise	8	Exercise for weight loss
*Trends related to technology:*	
		1	Wearable technology^2^
		20	Mobile exercise apps^2^
*Trends related to health:*	
		15	Exercise is medicine^2^
		16	Lifestyle medicine^2^

Note: ^1^appearance only in Iran, ^2^appearance only in the worldwide survey.

**Table 5.** A grouped comparative analysis of the top 10 fitness trends in Iran based on age, experience, career choice and annual salary.

**Sex**		**Age**		**Experience**		**Career choice**		**Annual salary**	
*Female (N = 227)*		*<35 years (N = 226)*		*<10 years (N = 191)*		*First Job (N = 330)*		*<$1,500 USD (N = 176)*	
Trend	Score	Trend	Score	Trend	Score	Trend	Score	Trend	Score
GExT	7.40 ± 1.90	STFW	7.31 ± 2.04	STFW	7.31 ± 2.00	STFW	7.28 ± 2.03	STFW	7.28 ± 1.99
DW	7.35 ± 2.04	ExWL	6.98 ± 2.06	GExT	6.93 ± 2.10	ExWL	7.03 ± 2.16	ExWL	7.06 ± 2.29
STFW	7.15 ± 2.03	GExT	6.91 ± 2.11	ExWL	6.82 ± 2.15	GExT	6.83 ± 2.06	DbW	6.76 ± 2.31
ExWL	7.02 ± 2.19	LcBG	6.56 ± 2.51	DbW	6.72 ± 2.32	LcBG	6.63 ± 2.48	GExT	6.72 ± 2.01
Pilates	6.77 ± 1.71	DbW	6.50 ± 2.45	LcBG	6.35 ± 2.41	DbW	6.62 ± 2.42	LcBG	6.63 ± 2.52
LcBG	6.71 ± 2.39	BWT	6.34 ± 2.20	BWT	6.35 ± 2.10	Out Act	6.20 ± 2.23	AquaEx	6.12 ± 2.44
BWT	6.54 ± 2.08	Pilates	6.21 ± 1.92	Pilates	6.27 ±1.77	BWT	6.17 ± 2.11	Out Act	6.12 ± 2.25
Core T	6.33 ± 2.05	Core T	6.03 ± 2.09	Core T	6.12 ± 2.02	Pilates	6.12 ± 1.99	Pilates	6.09 ± 1.91
Out Act	6.27 ± 2.29	Out Act	6.01 ± 2.19	AquaEx	5.98 ± 2.20	Core T	5.90 ± 2.14	BWT	6.04 ± 2.20
AquaEx	6.07 ± 2.33	AquaEx	5.97 ± 2.28	Out Act	5.95 ± 2.14	AquaEx	5.85 ± 2.32	Core T	5.99 ± 2.13
*Male (N = 181)*		*>35 years (N = 182)*		*>10 years (N = 217)*		*Second or Third Job (N = 78)*		*>$1,500 USD (N = 232)*	
STFW	7.25 ± 2.10	STFW	7.05 ± 2.07	STFW	7.09 ± 2.11	STFW	6.85 ± 2.15	STFW	7.07 ± 2.10
ExWL	6.75 ± 2.20	ExWL	6.80 ± 2.36	ExWL	6.96 ± 2.24	LcBG	6.71 ± 2.57	GExT	6.88 ± 2.12
LcBG	6.55 ± 2.62	LcBG	6.75 ± 2.48	LcBG	6.90 ± 2.54	GExT	6.56 ± 2.24	ExWL	6.78 ± 2.07
Out Act	6.04 ± 2.15	DbW	6.68 ± 2.39	GExT	6.65 ± 2.08	DbW	6.42 ± 2.45	LcBG	6.73 ± 2.48
GExT	5.99 ± 2.07	GExT	6.62 ± 2.07	DbW	6.45 ± 2.51	ExWL	6.30 ± 2.25	DbW	6.54 ± 2.44
DbW	5.61 ± 2.52	Out Act	6.37 ± 2.27	Out Act	6.36 ± 2.29	Pilates	6.10 ± 2.05	Out Act	6.23 ± 2.23
BWT	5.56 ± 2.16	Pilates	6.01 ± 2.10	Pilates	5.99 ± 2.17	Out Act	6.04 ± 2.22	BWT	6.15 ± 2.16
AquaEx	5.54 ± 2.31	BWT	5.81 ± 2.11	BWT	5.89 ± 2.21	Core T	5.96 ± 2.22	Pilates	6.15 ± 2.05
Core T	5.40 ± 2.18	Core T	5.77 ± 2.24	Core T	5.73 ± 2.26	BWT	5.83 ± 2.43	Core T	5.92 ± 2.15
Pilates	5.31 ± 2.04	AquaEx	5.67 ± 2.39	AquaEx	5.71 ± 2.44	AquaEx	5.76 ± 2.39	AquaEx	5.78 ± 2.24

Note: GExT: Group Exercise Training; DW: Dance-based Workouts; STFW: Strength Training (Free Weights); ExWL: Exercise for Weight Loss; LcBG: Low-Cost and Budget Gyms; BWT: Body Weight Training; Core T: Core Training; Out Act: Outdoor Activities; AquaEx: Aquatic Exercise. Scores are expressed as mean values ± standard deviation

## 4. Discussion

### 4.1. Summary of main findings

A web-based survey of health and fitness trends in Iran was conducted for the first time, aiming to help all industry stakeholders identify the current state of the health and fitness trends associated with specific services and programs. Furthermore, such a research attempt may support entrepreneurs, gym managers, practitioners and educators to strengthen customer engagement and experience through science-based strategies and applicable concepts in this evolving industry. In Iran, strength training with free weights, exercise for weight loss, group training, low-cost and budget gyms, dance-based workouts, outdoor activities, Pilates, body weight training, core training and aquatic exercise were identified as the top 10 health and fitness trends for 2024 (Table 2). Importantly, 75% of top 20 options were trends related to fitness activities associated with various exercise types and settings. Trends related to fitness modalities and specific populations were equally popular, having two trends each in the top 20. On the contrary, technology- and health-oriented trends demonstrated very low popularity nationwide, since no relevant trends were ranked among the top 20 most attractive selections. Interestingly, mind-body fitness modalities, such as Pilates and yoga were ranked in top 20, indicating high attractiveness in Iran. However, small group training (#22), fitness programs for older adults (#29) and circuit training (#33) showed poor popularity among respondents compared to other international surveys, given that these trends were not included in top 20 as recently observed in similar cross-sectional studies conducted in other countries and regions (Table 3).

### 4.2. What is most attractive?

The outcomes of the first-ever Iranian survey of health and fitness trends replicating the ACSM's methodology [28] indicate several similarities and differences with findings reported in other recently published surveys of this kind, aiming to detect trends in the health and fitness industry of numerous countries [34]–[42] and regions [29]–[33]. On average, strength training with free weights and exercise for weight loss were ranked #1 and #2 in the present study, respectively, which is a finding that does not fully agree with the results reported in other international surveys [28],[33]. These particular trends were also found as the number one and two selections in most subgroup analyses based on age, experience, career choice and annual salary, but not gender. Specifically, females ranked strength training with free weights #3 compared to males (#1) in Iran. However, strength training with free weights seems to be an attractive area for fitness businesses and practitioners working with clients in various exercise settings, given that this trend is currently included in the top health and fitness trends in Australia (#3), Brazil (#6), China (#9), Europe (#11), Mexico (#11), the United States (#2) and worldwide (#2) [28],[32],[33]. Strength training with free weights is a traditional fitness activity; however, it appears popular not only in Iran but also globally. In the attempt to explain the popularity of this particular trend from a scientific standpoint, the existing body of evidence indicates that resistance training programs may be a safe and effective exercise approach for the masses, targeting numerous training goals and inducing important psychophysiological benefits in various real-world exercise settings [43].

### 4.3. Qualified fitness professionals: Are they key players?

Trends related to fitness professionals such as licensure for fitness professionals (#12) and employing certified fitness professionals (#21) appear moderately attractive in Iran, since the former was more popular than the latter for 2024 and both were not ranked among the top 10 health and fitness trends nationwide. In contrast to this observation, this category of trends has been reported popular in other international surveys [28],[33], highlighting some key differences among the status of the Iranian health and fitness industry and other national ones in terms of the eligibility criteria aspiring practitioners should meet before entering the industry. However, 88% of participants in the present study held a bachelor's degree in exercise science or a related field and 66% held an additional relevant postgraduate degree (Table 1). This is an important observation underlining the culture of local practitioners and the current state of the informal job requirements for those involved in the health and fitness industry, even though the fitness profession is not regulated nationwide and higher education is not obligatory for fitness professionals in Iran. It is noted that the high demand for qualified training personnel is systematically increasing due to the rapidly growing prevalence of people impacted by the most common cardiometabolic health disorders that adversely influence physical function, quality of life and life expectancy worldwide [6],[10]. Given that a significantly high percentage of the adult population in Iran are not considered apparently healthy individuals, well-equipped professionals appear to be a priority nationwide in order to tackle the inactivity and obesity epidemics. Relevant academic education may be a great tool for practitioners seeking to work in the field and supporting various populations by offering client-centered fitness services based on proven exercise strategies. Thus, regulation and licensure requirements as well as advanced professional credentials for practitioners, which were ranked as promising trends in the present study, may also protect public health and raise the exercise profession through higher standards locally. Additionally, qualified fitness professionals should be a part of a multidisciplinary client healthcare team as extensively reported, aiming to promote an inclusive environment in various exercise settings [44]–[47].

### 4.4. What is trending in fitness activities?

Interestingly, almost 80% of the Iranian top trends were related to various fitness activities. In particular, strength training with free weights, low-cost and budget gyms, dance-based workouts, outdoor activities, Pilates, bodyweight training, core training and aquatic exercise were reported as the most popular. It is worth mentioning that some fitness activities (e.g., low-cost and budget gyms, dance-based workouts, aquatic exercise, licensure for fitness professionals, resistance band training, walking/running/cycling clubs and stretch-based training) included in the top 20 trends in Iran were not ranked among the most popular in other countries and regions (Table 3). Additionally, dance-based workouts were selected as the fifth (second among females) most popular trend in the present study (Table 5), which is not in agreement with data observed in all available international surveys. On the other side, circuit training and small group training were attractive options among international respondents based in Australia, Brazil, Europe, Mexico, the United States and worldwide, but not in Iran (Table 3). These observations cannot be thoroughly explained here, since both low-cost and budget gyms (#4) and boutique fitness studios (#11) are present on the top trends list in Iran. However, the high ranking of more traditional fitness activities, such as strength training and group training than those primarily associated with first-line services in boutique gyms and personal training studios may support the reasons behind the current status of these particular trends in the Iranian health and fitness industry, Importantly, targeted studios are an emerging fitness facility type in mature and developed markets [1],[2],[48],[49] and has been reported as one of the most promising workplaces among European exercise professionals [50]. In general, this exercise setting appears to have great potential in the future, especially in emerging markets seeking to invest in more innovative and targeted fitness services and programs, aiming to increase customer engagement, satisfaction, loyalty and retention [49],[51]. Importantly, muscle-strengthening and aerobic-based activities were reported as popular in the present survey, which is a finding corroborates the main results recently found not only in the worldwide [28] but also in various national and regional studies [29]–[33]. Moreover, nontraditional fitness activities, such as body weight training (#8), core training (#9), functional fitness training (#17) and high-intensity interval training (#18) tend to be attractive in Iran, which is a finding supported by current evidence showing that multicomponent fitness programs incorporating various high ranked activities into a single workout may be feasible, effective, time-efficient and pleasant exercise strategy in the real world [52]–[54]. Another important result from the Iranian survey that should be taken into account is the high ranking of mind-body fitness trends, such as Pilates (#7) and yoga (#13). These particular trends, especially Pilates, were not always present in the top 20 health and fitness trends among other countries and regions (Table 3) and this is a remark that may need further investigation in the future. However, such alternative fitness activities have been established as some inclusive and effective exercise approaches among consumers and practitioners worldwide, promoting beneficial psychophysiological adaptations [55]–[57].

### 4.5. Which setting is most popular? Private, semi-private or group training?

Group training has been selected as the most popular training modality (#3) whereas personal (#19) and small group (#22) training were ranked lower in Iran for 2024. Such an outcome is not in line with the results recently reported by other international surveys [28],[33]. According to the available data, private setting is clearly more promising than semi-private or group setting internationally (Table 3). Fitness markets from countries characterized by low socioeconomic status are commonly struggling with the personal and small group training services, since personalized training programs are more expensive compared to self-paced or group-based training options [58],[59]. Thus, traditional and unsupervised fitness activities, such as strength training with free weights and group training seem to be more attractive in Iran. In contrast, private and semi-private training settings are on the rise in Europe and worldwide [1],[48], shaping the future of the health and fitness industry through more engaging and client-centered exercise concepts, aiming to deliver unique fitness experiences in a new industry landscape [60]. Interestingly, the profession of personal trainer has been documented as the second most promising occupational role in Europe [50],[61]. In this study, on average, personal training ranked lower (#19) than in other international surveys where this particular trend has been included in top 10 (Table 3). Such a finding may be supported by the fact that Iran is not a developed fitness market investing in customized exercise solutions for the big majority of consumers. Moreover, health/wellness coaching shows low popularity nationwide and this is an observation linked to the fact that health-related trends were totally absent from top 20 in Iran for 2024 compared to other countries/regions (Table 3) and globally (Table 4).

### 4.6. What is less popular?

Although the vast majority of adults in Iran have been impacted by inactivity, overweight/obesity and several obesity-related chronic diseases [10], no trends related to health were ranked among the top 20 options nationwide (Table 4). However, exercise for weight loss has been reported as the second most popular trend in the present survey, indicating the high demand of fitness services with a focus on people living with excess weight. Moreover, this particular trend along with children and exercise (#14) were the only trends related to programs oriented to specific populations included in the top 20 Iranian health and fitness trends (Table 4). Notably, older adults do not tend to be an attractive clientele in Iran compared to the international level where fitness programs for older adults have been documented as one of the most popular health and fitness trends (Table 3). However, exercise for weight loss appears to be a promising space for business not only in Iran but also worldwide, demonstrating an exceptional attractiveness to industry stakeholders in several countries and regions [28],[33]. On the other side, health-related fitness services do not currently demonstrate strong potential for growth and innovation, since several relevant trends (e.g., post-rehabilitation classes, exercise is medicine, lifestyle medicine and clinical integration/medical fitness) were not reported as popular in Iran compared to other national, regional and global surveys (Table 3).

Notably, the powerful role of technology in the evolution of the health and fitness industry worldwide has been well documented, showing that social media, mobile apps, online services and state-of-the-art equipment can be significantly impactful factors for developing new health and fitness trends worldwide [62]. However, technology-oriented trends such as wearable technology, outcome measurements, online personal training, mobile exercise apps, virtual reality exercise training, online live and on-demand exercise classes and electrical muscle stimulation training were not ranked among the top 20 in Iran. This finding agrees with the results reported in several international surveys investigating national [34]–[42] and regional data [29]–[33], but not global ones [28] (Table 3). According to the present results, technology-based services do not appear a top priority in the Iranian health and fitness industry, since digital fitness services, programs and products do not demonstrate an impactful role in developing health and fitness trends nationwide. This observation is in line with the main outcomes published by other similar surveys, investigating the top trends at the international level (Table 3), showing that health and fitness trends related to technology are not yet broadly recognized among industry stakeholders. This remark cannot be explained here, and therefore further investigation is warranted in this particular research area, aiming to detect the potential factors behind the minor role of technology in launching new trends in the Iranian health and fitness industry. In summary, technology advances and digital transformation are constantly growing within the health and fitness industry worldwide [63]. Consequently, industry stakeholders, such as gym operators/managers, fitness professionals and educators should put more attention to the importance of utilizing digital services, since such a strategy may increase user communication and help consumers engage in unique and more interactive fitness experiences [64],[65].

### 4.7. The impact of COVID-19 experiences

Taking into account that the global health and fitness industry has been remarkably impacted by the COVID-19 pandemic in the past few years [1],[48], the interaction between technology and fitness was stronger than ever throughout an almost 3-year global epidemiological crisis [48]. However, the rapid digitalization and aggressive transformation of the entire health and fitness industry aiming to survive under an unprecedented business environment, it was an unpredictable situation that promoted nontraditional fitness services mainly carried out in a virtual gym environment. Thus, outdoor activities gained a lot of popularity during the COVID-19 pandemic due to fitness club restrictions worldwide [27],[32]. However, this particular trend appears to retain its attractiveness even after that challenging period, since it has been ranked among the top 10 health and fitness trends in several international surveys (Table 3). In Iran, outdoor activities have been selected as the sixth most popular option, showing that such fitness activities are still attractive nationwide. This finding may be interacted with another one with respect to the popularity of walking, running and riding (#16). Both trends are considered accessible, cost-effective and pleasant fitness activities, and therefore their popularity may be linked to the fact that unsupervised and user-friendly exercise approaches are attractive among consumers and practitioners in Iran. Furthermore, the running movement supported by recreational exercisers creating a global community may be an additional factor positively affecting the popularity of this particular trend at the national level [66]. On the other hand, group training had been meaningfully influenced by the COVID-19 period due to rigorous hygiene protocols and restrictions, and thus it demonstrated low ranking on the top 20 health and fitness trends list worldwide in 2020–2022 [27],[31],[32]. Given that exercisers were discouraged to engage in group-based fitness activities in gym facilities for almost three years, group training may be now a dynamic trend attracting the masses seeking to live again some engaging exercise experiences in a group-style exercise setting.

### 4.8. Strengths and limitations

The present cross-sectional survey, as an observational study, has numerous weaknesses, such as difficulties in determining causal effects, cohort differences and potential report biases. More specifically, the lack of randomization in the sampling process and potential coverage errors seem to be the main drawbacks of this study. Moreover, the absence of financial or material incentive to respondents may be an additional strategy for collecting more data in a survey of this kind. On the other hand, the replication of the ACSM's methodology broadly used in similar international surveys appears to be the main strength of the present study, underlining a high degree of standardization that may reduce potential bias frequently observed in this type of cross-sectional studies. The fact that respondents were based in all provinces of the country provides important summarized data for Iran which may be an additional strength of this national survey. However, a larger sample size would be a positive point in a future research attempt at the national level. With the dissemination of the present outcomes, Iran is now included in a list of countries and regions that regularly carry this type of surveys, allowing remarks to be reported and compared over time. This cross-sectional survey may play some role in the evolution of services and products in the Iranian health and fitness industry. Hence, it may be important to continue with this research project nationwide in the coming years.

## 5. Conclusions

The outcomes from the first-ever cross-sectional study emphasized on health and fitness trends in Iran may help the industry stakeholders in making critical business and professional development decisions. On average, strength training with free weights was the most popular trend nationwide for 2024. This result is not fully aligned with current outcomes from other recently published international surveys. This national survey provides the opportunity to examine important comparisons among Iran and other countries or regions, aiming to deliver remarks with respect to the most popular health and fitness trends linked to numerous fitness services, products and programs globally. Given that various lifestyle-related chronic diseases are currently on the rise not only in Iran but also worldwide, the purpose of this national survey was to guide entrepreneurs, practitioners, educators and customers highly involved in the Iranian health and fitness industry on how to advance and apply safe, effective and enjoyable fitness services related to the top industry trends. Considering that the evolving development of the health and fitness industry requires gym operators and managers as well as exercise professionals to be constantly updated and to use science-based practices within a multifaceted and competitive space, the interpretation of the present findings into client-centered services may support positive exercise experiences in the health and fitness industry in Iran. Further study should certainly help the examination in this particular research area, aiming to detect whether clients' opinions vary from the present outcomes in Iran and countries or regions. Such a future research attempt may promote the important mission of the health and fitness industry, aiming to spread the message that regular exercise should be a high priority for the masses engaging in novel and science-based fitness services.
